# 1692. Increasing incidence of *Streptococcus anginosus* group intracranial complications of sinusitis, otitis media and mastoiditis in a pediatric population

**DOI:** 10.1093/ofid/ofad500.1525

**Published:** 2023-11-27

**Authors:** Elisabeth Hoyer, Marritta Joseph, James Dunn, Howard Weiner, Amy Dimachkieh, Anthony R Flores, Sheldon L Kaplan, Jesus G Vallejo, Jonathon C McNeil

**Affiliations:** Baylor College of Medicine, Houston, Texas; Baylor College of Medicine, Houston, Texas; Texas Children's Hospital, Houston, TX; Baylor College of Medicine, Houston, Texas; Baylor College of Medicine, Houston, Texas; University of Texas Health Sciences at Houston, Houston, Texas; Baylor College of Medicine, Houston, Texas; Baylor College of Medicine, Houston, Texas; Baylor College of Medicine, Houston, Texas

## Abstract

**Background:**

The *Streptococcus anginosus* group (SAG) pathogens (*S. anginosus, S. intermedius,* and *S. constellatus)* are oral flora with the potential to cause head and neck space infections including brain abscesses. Several centers noted an increase in intracranial abscesses in children in the SARS-CoV-2 era, prompting a CDC health alert in May 2022. Notably, a large proportion of such reported cases were associated with SAG and/or sinusitis. We examined the epidemiology of pediatric intracranial abscesses with a focus on SAG in the pre- and post-pandemic periods.

**Methods:**

Cases of intracranial abscesses (parenchymal, subdural, epidural) of any microbiologic etiology from January 1, 2011 to December 31, 2022 were identified at Texas Children's Hospital by ICD10 codes. Subjects were cross referenced with culture results of the microbiology laboratory. Cases included were those associated with either otitis media, mastoiditis, or sinusitis. Temporal trend in these infections was investigated with linear regression.

**Results:**

157 cases were identified and 59.9% were due to SAG. The incidence of all sinogenic/otogenic intracranial infections (p=0.006), and SAG specific infections (p=0.01), increased from 2011 to 2022 (**Figure 1**). SAG infection was more often associated with multiple surgeries and these subjects were specifically more likely to require craniotomy or craniectomy (**Figure 2**). Among sinogenic abscesses, *S. intermedius* was the most common pathogen while among otogenic cases *S. pyogenes* predominated (**Figure 3**). There were 8 cases of craniectomies and one relapse (due to SAG) after replacement of autologous bone flap. After March 2020, 6/46 cases tested positive for SARS-CoV-2 (13%); the severity of infection was not significantly different with concomitant COVID and intracranial abscess. The proportion of SAG-associated infections was similar prior to and after the COVID pandemic.

**Figure:**
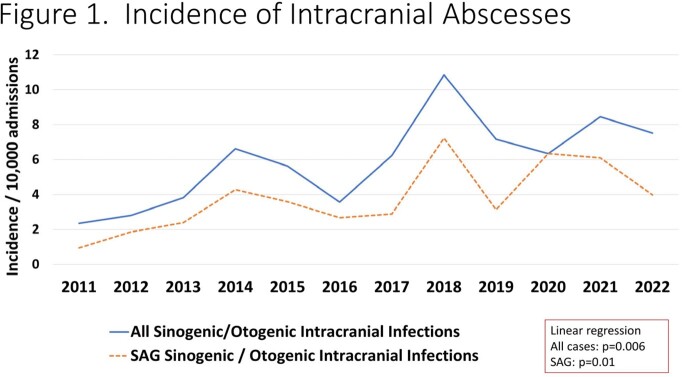

**Figure:**
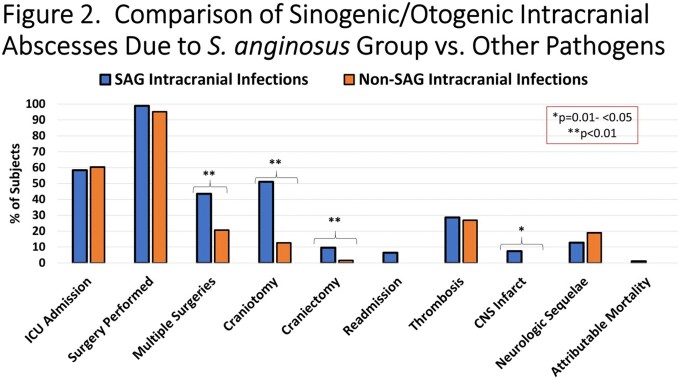

*- p= 0.01- <0.05 ** p< 0.01

**Figure:**
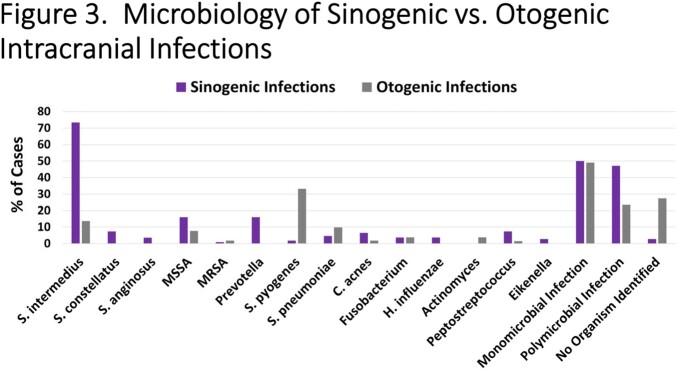

**Conclusion:**

Over the last decade, intracranial complications of sinusitis/otitis have been increasing, and specifically those caused by SAG. This observed trend clearly predated the COVID pandemic. SAG cases were associated with greater need for surgical intervention and specifically neurosurgery. Further work is necessary to determine the explanation for these rising infections.

**Disclosures:**

**Sheldon L. Kaplan, MD**, MeMed: Grant/Research Support|Pfizer: Grant/Research Support|Pfizer: Honoraria **Jonathon C. McNeil, MD**, Allergan: Grant/Research Support|Nabriva Therapeutics: Grant/Research Support

